# Brain imaging before primary lung cancer resection: a controversial topic

**DOI:** 10.3332/ecancer.2017.749

**Published:** 2017-06-20

**Authors:** Zoe Hudson, Eveline Internullo, Anthony Edey, Isabel Laurence, Davide Bianchi, Alfredo Addeo

**Affiliations:** 1 Bristol Cancer Institute, University Hospital Trust, Horfield Road, Bristol BS2 8ED, UK; 2 Cardio-thoracic Unit, University Hospital Trust, Horfield Road, Bristol BS2 8ED, UK; 3 Radiology Department, Southmead Hospital, North Bristol Trust, Southmead Rd, Westbury-on-Trym, Bristol BS10 5NB, UK; 4 Reseau Santé Balcon du Jura, Rue des Rosiers Sainte-Croix, Vaud 1450, Switzerland

**Keywords:** lung surgery, brain imaging, MRI

## Abstract

**Objective:**

International and national recommendations for brain imaging in patients planned to undergo potentially curative resection of non-small-cell lung cancer (NSCLC) are variably implemented throughout the United Kingdom [Hudson BJ, Crawford MB, and Curtin J *et al* (2015) **Brain imaging in lung cancer patients without symptoms of brain metastases: a national survey of current practice in England** *Clin Radiol* https://doi.org/10.1016/j.crad.2015.02.007]. However, the recommendations are not based on high-quality evidence and do not take into account cost implications and local resources. Our aim was to determine local practice based on historic outcomes in this patient cohort.

**Methods:**

This retrospective study took place in a regional thoracic surgical centre in the United Kingdom. Pathology records for all patients who had undergone lung resection with curative intent during the time period January 2012–December 2014 were analysed in October 2015. Electronic pathology and radiology reports were accessed for each patient and data collected about their histological findings, TNM stage, resection margins, and the presence of brain metastases on either pre-operative or post-operative imaging. From the dates given on imaging, we calculated the number of days post-resection that the brain metastases were detected.

**Results:**

585 patients were identified who had undergone resection of their lung cancer. Of these, 471 had accessible electronic radiology records to assess for the radiological evidence of brain metastases. When their electronic records were evaluated, 25/471 (5.3%) patients had radiological evidence of brain metastasis. Of these, five patients had been diagnosed with a brain metastasis at initial presentation and had undergone primary resection of the brain metastasis followed by resection of the lung primary. One patient had been diagnosed with both a primary lung and a primary bowel adenocarcinoma; on review of the case, it was felt that the brain metastasis was more likely to have originated from the bowel cancer. One had been clinically diagnosed with a cerebral abscess while the radiology had been reported as showing a metastatic deposit. Of the remaining 18/471 (3.8%) patients who presented with brain metastases after their surgical resection, 12 patients had adenocarcinoma, four patients had squamous cell carcinoma, one had basaloid, and one had large-cell neuroendocrine. The mean number of days post-resection that the brain metastases were identified was 371 days, range 14–1032 days, median 295 days (date of metastases not available for two patients).

**Conclusion:**

The rate of brain metastases identified in this study was similar to previous studies. This would suggest that preoperative staging of the central nervous system may change the management pathway in a small group of patients. However, for this group of patients, the change would be significant either sparing them non-curative surgery or allowing aggressive management of oligometastatic disease. Therefore, we would recommend pre-operative brain imaging with MRI for all patients undergoing potentially curative lung resection.

## Introduction

Despite many advances in the management of non-small-cell lung cancer (NSCLC) in recent years prognosis still remains limited and surgery does not result in long-term cure for all patients. Improved selection of patients for surgical intervention is essential. There is guidance on brain imaging as part of the pre-operative work up from many international groups. However, it is acknowledged that within the UK individual centre practice is very variable [1].

The American College of Chest Physicians (ACCP) guidelines recommend magnetic resonance imaging (MRI) of the brain for patients with clinical stage III or IV disease with or without symptoms of intracranial disease [2], while the National Institute for Health and Care Excellence (NICE) [3] and the British Thoracic Society (BTS) guidelines [4] recommend consideration of MR or contrast-enhanced CT of the head in patients selected for treatment with curative intent, particularly in stage-III disease. The level of evidence to support these recommendations is low.

We ran a study to firstly establish the proportion of patients who developed brain metastases following curative surgery, all of whom were staged with preoperatively with CT chest abdomen and pelvis (CT CAP) and PET–CT and underwent post-operative CT CAP as part of routine follow-up within our centre. As brain imaging was not routinely performed pre-operatively unless the patient was symptomatic, we also quantified within this study how many brain metastases could have been detected by preoperative MRI at the time of surgery.

## Materials and methods

### Study population

Consecutive patients who underwent curative surgical resection of biopsy-proven NSCLC from January 2012–December 2014 in a single tertiary centre thoracic surgery unit were included in this study. As part of their diagnostic work up, all patients had undergone PET-CT scanning alongside CT CAP. All patients had been discussed at a regional multidisciplinary meeting (MDT). Patients who had undergone surgical resection but did not have accessible radiological images were excluded from the study.

### Covariate definitions

TNM stage as per TNM 7 [5] and histological subtype were obtained from pathological reports. Local and regional electronic radiology systems were used to determine whether the patients had undergone imaging of the brain pre- or post-operatively. The presence or absence of brain metastases was noted along with the date of the scan confirming or refuting their presence. If clinical details were available as to whether the patient was symptomatic or not, these were also recorded (Table 1).

### Data collection and statistical analysis

Data were gathered retrospectively and recorded electronically. Statistical formulae inbuilt into the programme were then used for analysis.

In order to determine whether the brain metastasis might have been detectable on a pre-operative MR brain, we estimated how large a metastasis would have been at the time of surgery. This was calculated by using the measurements of the largest metastasis and the time from the date of surgery and the change in diameter of lesions over time using this volume doubling time (rounded to 60 days), assuming the metastases were spherical based [6]. We plotted the change in diameter over time of 2 mm and 5 mm diameter lesions and then plotted the maximum tumour diameter measured on the diagnostic scan against the number of days after surgery that the imaging was performed and the data of surgery was time 0. The lesions that fell above the detection limit curves were classified as likely visible had a pre-operative MRI been performed.

## Results

### Frequency of brain metastases

Five hundred eighty-five patients, who had undergone curative resection of their NSCLC over the time period stated earlier, were identified. Of them, 471 had electronically accessible radiology records. The remaining 114 (20%) patients did not have accessible radiology record as they had been referred from distant centres where the imaging was stored but not available to for central review.

Twenty-five out of 471 (5.3%) patients had radiological evidence of brain metastases identified either pre-operatively or post-operatively. Five (1.1%) patients presented with symptoms that led to brain imaging diagnosing metastatic disease but had otherwise operable NSCLC (Table 2). These patients underwent definitive treatment for their solitary brain metastasis and then underwent resection of the lung primary tumour.

One patient had been diagnosed with both NSCLC and colorectal adenocarcinoma; on review of histology, it was felt that the brain metastasis was more likely to be related to the colon cancer. One patient had been radiologically diagnosed with a brain metastasis, while all the clinical evidence supported a diagnosis of cerebral abscess, which was drained and histologically confirmed not to be malignant. This left a total of 18 (5%) patients who developed brain metastases in the post-operative period.

### Clinicopathological information

Of the 18 patients post-operatively diagnosed with brain metastases the mean number of days to diagnosis was 271 (range 14–1032, median 295 days). Nine presented within 12 months of their resections and, of these 9, 5 presented within 6 months. Of the nine patients presenting within one year of their resection, four had no evidence of systemic relapse, two had local and loco-regional nodal relapse. No data were available for three patients. Of those who had CNS, only relapse two were able to receive stereotactic radiosurgery (SRS); for the other two patients, no data were available

### Imaging modality for detection

Pre-operative brain MRI has proven to be superior to CT in detecting brain metastasis in potentially operable lung cancer patients [7–9]. Despite this, MRI is a more expensive and time-consuming method and a limited resource in the UK Standard practice in all patients considered for surgery is PET-CT and this offers a further opportunity for the detection of cerebral metastases. Hjorthuag *et al*. carried out a large study to assess the feasibility of this approach [10] founding that the sensitivity of PET-CT for diagnosing brain metastases was greater than 70%, with similarly high specificity. They suggest that PET-CT could be used as a screening tool and further imaging arranged if abnormalities are detected.

**Figure 1.**
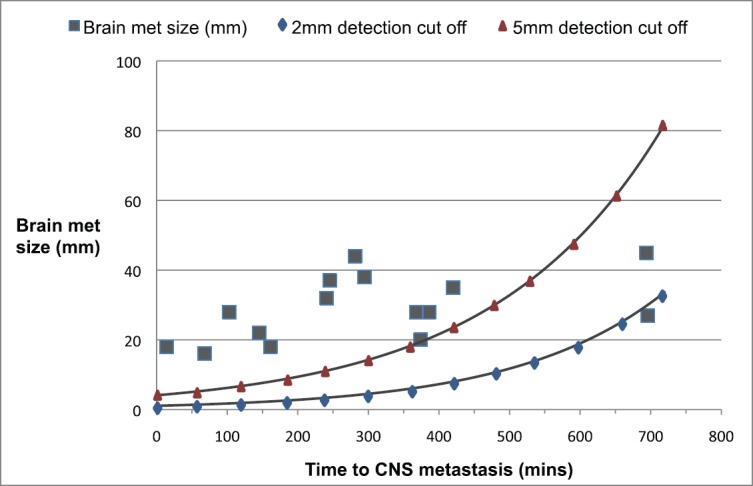
Plots of size of brain metastases and time of diagnosis after surgery with 2 and 5 mm cut-off.

As previously explained, in order to determine whether the brain metastasis might have been detectable on a pre-operative MRI brain, we estimated how large a metastasis would have been at the time of surgery. We decided to exclude three patients from this portion of the analysis, one as no information available on timing of the brain metastases, two as they developed metastases at over 2 years post-surgery, and we felt that these lesions could not reasonably have been detected with a pre-surgery MRI brain. We projected in Figure 1 the plot of the remaining 15 patients with curves to show both 2 mm and 5 mm detection thresholds. Using a 5 mm detection threshold, 12 (66%) of brain metastasis should have been of at least 5 mm maximum diameter at time 0. Using a 2 mm detection threshold, 14 (80%) would have been at least 2 mm in maximum diameter at time 0. These would be potentially detectable had MRI brain been performed as part of the staging process. This means that 3.3% (12/366) of our patients who underwent surgery would have had their brain metastases detected by pre-operative MRI and this might have been as high as 3.8% (14/366) with optimal MRI detection limits

## Discussion

ACCP guidelines recommend MRI of the brain in those with clinical stage-III or stage-IV disease even if neurologically asymptomatic, while NICE and BTS guidelines recommend consideration of MRI or contrast-enhanced CT of the head in patients selected for treatment with curative intent, particularly in stage-III disease. The level of evidence to support these recommendations is low, owing to there being no large study to definitively support this guidance or a clear consensus of opinion. The widespread use of PET–CT preoperatively, which often includes head CT, may also detect a proportion of brain metastases. Thus, the potential gain from more sensitive brain imaging, such as MRI or contrast-enhanced CT of the brain, is uncertain. Furthermore, recent American guidelines have suggested not offering brain imaging to patients with early-stage disease due to a low incidence of brain metastases identified when patients with stage IA tumours were imaged as part of their diagnostic work up [11]. However, of 18 patients identified in our study, four had pathological stage-IA disease at the time of surgery, these patients generally had a longer interval from surgery to detection. However, two patients with stage-1A disease presented with brain metastases within 12 months and one of these within 6 months. Those who subsequently developed metastases were more likely to have had an adenocarcinoma resected and the majority had early-stage malignancy. International guidelines agree in considering additional brain imaging, especially in those with stage-III disease without any strong evidence to support it [12, 13]. While proportionally more stage-III patients are considered to be at higher risk of developing brain metastases, our finding shows the opposite and pre-operative brain imaging should be applied irrespective of stage.

Overall, the rate of detection of brain metastases within our study was 5%, 25 patients in total, with 9/471 (1.9%) of patients developing brain metastases within 12 months of their surgery. Similar frequencies have been described in other studies [14]. Interestingly, within our study, 80% of the brain metastasis could have been detected by using a high sensitivity (2 mm cut-off) MRI brain at the time of surgery (time 0). For these patients, detecting secondary brain lesion pre-operatively could have several implications: firstly, the majority of patients had undergone an extensive operation that had proved non-curative due to the presence of metastatic disease at presentation. Secondly, for a small proportion of patients, the detection of their metastatic disease, particularly if oligometastatic disease within the central nervous system (CNS) allowed more aggressive treatment options in the form of surgery or stereotactic radiotherapy to the brain metastases. There is in fact growing evidence that resection of brain metastases improves survival outcomes in tumours with low-volume extracranial cancer [11, 15] with five-year survival rates up to 21% for patients who have had radical treatment of their brain metastases either with surgery or with stereotactic radiotherapy. The use of MRI brain to detect occult brain metastases provides not only an opportunity to treat oligometastatic disease, but studies have also reported improved survival in those with a good performance status at the time of diagnosis [15]. MRI brain may be replaced in the future by MRI-PET or new generation PET-CT which would come with better imaging resolution and would allow us to comprehensively stage the patient with a single radiological investigation.

Our study is in line with other similar findings [14] which lend further weight to the validity of our findings.

We acknowledge some limitations within the study: the calculation of growth curves is a potential limitation of this study as our volume doubling time estimate was based on the best evidence in the literature, but we have also assumed exponential growth. A second related limitation is that we have assumed the metastases to be spherical. This is clearly not the case and metastases may grow more in one dimension than another, although we observed that the majority of metastases in our and other studies were approximately spherical. We accept that the MRI scan cut-offs of 2 mm and 5 mm are arbitrary and that some centres may be able to detect lesions below this threshold. We chose these numbers after discussion with consultant neuroradiologists based at our institution. Further limitation of the study is the fact that we could access 471 radiological records of the 585 patients, which means we could not analyse 20% of the radiological records. This was meanly caused by the fact that some of the lung cancer patients that undergo surgery within our centre, had their subsequent follow-up locally with no radiological record available within our centre.

## Conclusions

Based on our findings, we suggest that patients should have pre-operative MRI brain before undergoing surgery with curative intent in NSCLC regardless of pre-operative stage, especially in those with adenocarcinoma histology. Our study confirms that the vast majority of the brain metastases could have been detected if preoperative brain MRI had been performed, similarly to other experiences and provides evidence to change the current recommendations to ensure that patients receive optimal staging before planning radical treatment.

The study did not include a sufficient number of patients to explore prognostic factors in depth and a detailed cost-effectiveness analysis is warranted prior to implementation in routine clinical practice.

## Figures and Tables

**Table 1.** Patients’ characteristics.

Patient	Pre-op staging (TNM)	Pathological staging (TNM)	Pathological staging group (AJCC)	Histology	Time to CNS metastasis (days)	Size mm
1	T3 N0 M0	pT2b Nx Mx R0	IIB	Squamous cell	1032	30 mm
2	T4N0M0	pT4 pN0 Mx R0	IIIA	Adenocarcinoma	696	27 mm
3	T1bN0M0	pT1b pN0 MX R0	IA	Adenocarcinoma	420	35 mm
4	T2bN2M0	pT2b pN0 Mx R0	IIIA	Basaloid	374	20 mm
5	T2aN2M0	pT3 pN0 Mx R0	IIB	Squamous cell	241	32 mm
6	T1bN0M0	pT1a Nx Mx R0	IA	Adenocarcinoma	694	45 mm
7	Not available	pT3 pN1 MX R0	IIIA	Adenocarcinoma	14	18 mm
8	T2bN0M0	pT2b pN1 MX R0	IIB	Adenocarcinoma	68	16 mm
9	T3N1M0	pT3 pN1 MX R0	IIIA	Adenocarcinoma	295	38 mm
10	T3N2M0	pT3 pN1 MX R0	IIIA	Adenocarcinoma	245	37 mm
11	Not available	pT3 pN1 MX R0	IIIA	Squamous cell	Not available	28 mm
12	T2aN0M0	pT2a, pN0 Mx R0	IB	Adenocarcinoma	103	28 mm
13	T1aN0M0	pT1a pN0 MX R0	IA	Large cell neuroendocrine	281	44 mm
14	T2bN0M0	pT3 pN1 Mx R0	IIIA	Adenocarcinoma	788	32 mm
15	T3N0M0	pT3 pN0 Mx R1	IIB	Squamous cell	145	22 mm
16	T1bN0M0	pT3 pN0 MX R0	IIB	Adenocarcinoma	386	28 mm
17	T2aN1M0	pT1b pN0 pMX R0	IA	Adenocarcinoma	161	18 mm
18	T1bN0M0	pT2a pN1 pMX R0	IIA	Adenocarcinoma	368	16 mm

**Table 2.** The presence of symptoms and systemic relapse (all patients).

	Yes	No	Not known
Symptoms	12	0	6
Systemic relapse	4	10	4
